# Supplementary material for: Genetically prolonged beige fat in male mice confers long-lasting metabolic health
Source: Nat Commun. 2023 May 12;14:2731. doi: 10.1038/s41467-023-38471-z (PMC10175245; doi:10.1038/s41467-023-38471-z)
Supplement: Supplementary file 1 — Supplementary Information [file 41467_2023_38471_MOESM1_ESM.pdf]

Supplementary Material

Supplementary Figures and Tables

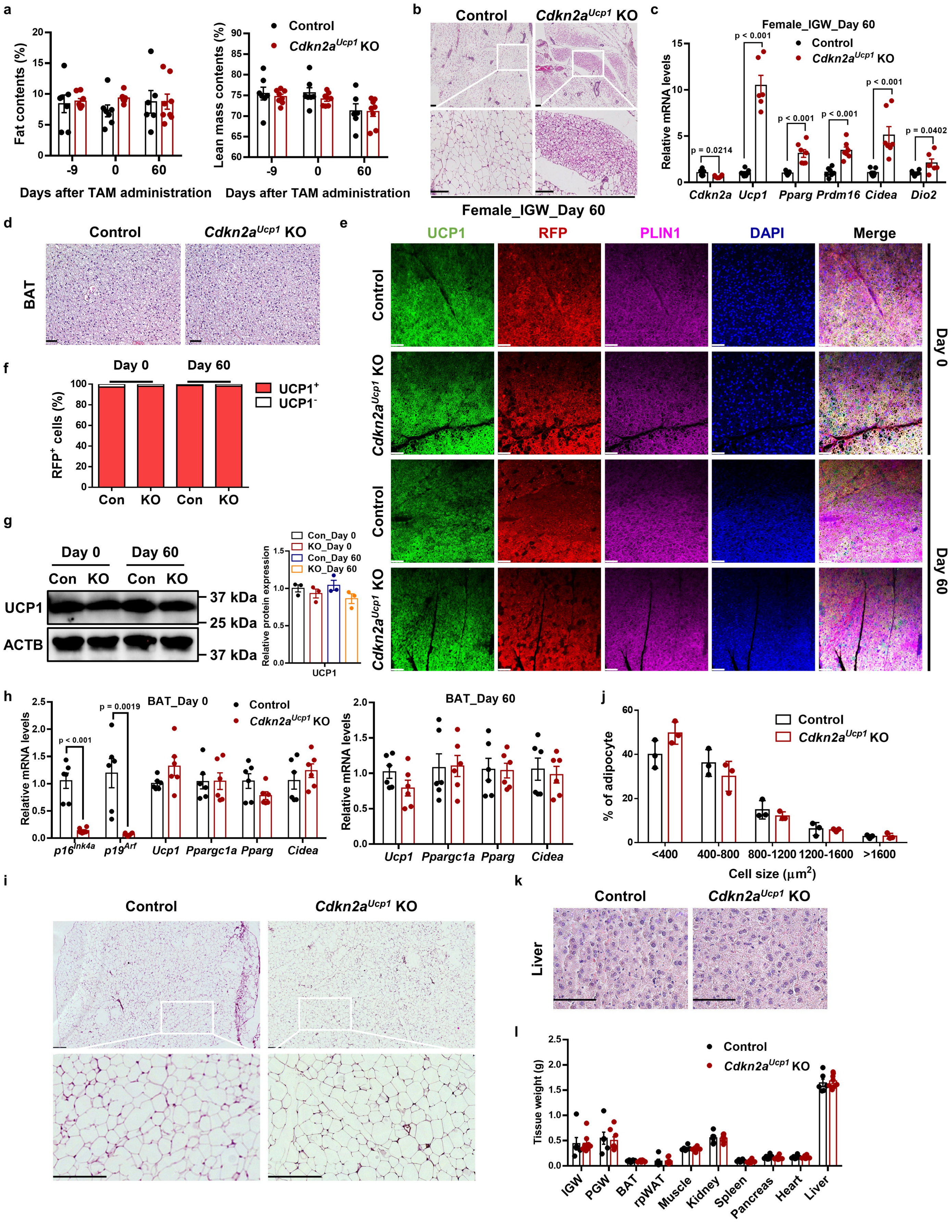

a
Fat contents (%)
Lean mass contents (%)
Control
Cdkn2a^Ucp1 KO
Days after TAM administration
b
Female_IGW_Day 60
c
Female_IGW_Day 60
Relative mRNA levels
p = 0.0214
p < 0.001
p = 0.0402
Cdkn2a Ucp1 Pparg Prdm16 Cidea Dio2
d
BAT
e
UCP1 RFP PLIN1 DAPI Merge
Day 0
Day 60
f
RFP+ cells (%)
UCP1+
UCP1-
Con KO Con KO
g
UCP1
ACTB
37 kDa
25 kDa
Relative protein expression
Con_Day 0
KO_Day 0
Con_Day 60
KO_Day 60
h
BAT_Day 0
BAT_Day 60
p = 0.0019
p16^Ink4a p19^Arf Ucp1 Ppargc1a Pparg Cidea
i
j
% of adipocyte
Cell size (μm^2)
<400 400-800 800-1200 1200-1600 >1600
k
Liver
l
Tissue weight (g)
IGW PGW BAT rpWAT Muscle Kidney Spleen Pancreas Heart Liver

Figure Legend

Supplementary Fig. 1: Knockout of *Cdkn2a* does not affect BAT characteristics after the rewarming period following cold exposure.

a, NMR analysis of fat (left panel) and lean masses (right panel) of body weight of control or $Cdkn2a^{Ucp1}$ KO male mice fed a chow diet.

b, Tile scan of IGW from control or $Cdkn2a^{Ucp1}$ KO female mice at day 60 post withdrawal of cold stimulus. Scale bar, 100 μM.

c, qPCR analysis of the mRNA expression of *Cdkn2a* and thermogenic genes in IGW from control or $Cdkn2a^{Ucp1}$ KO female mice at day 60 post withdrawal of cold stimulus.

d, Representative H&E staining images of BAT from control or $Cdkn2a^{Ucp1}$ KO male mice. Scale bar, 100 μM.

e, Immunofluorescence staining of UCP1 and RFP in BAT from control or $Cdkn2a^{Ucp1}$ KO male mice. Scale bar, 100 μM.

f, Quantification of the percentage of RFP^+ cells that express endogenous UCP1.

g, Western blot analysis of UCP1 in BAT from control or $Cdkn2a^{Ucp1}$ KO male mice (n = 3).

h, qPCR analysis of mRNA expression of thermogenic genes in BAT from control or $Cdkn2a^{Ucp1}$ KO male mice at day 0 (left panel) and day 60 (right panel) post withdrawal cold stimulus.

i, Tile scan of PGW from control or $Cdkn2a^{Ucp1}$ KO male mice. Scale bar, 100 μM.

j, Quantification of adipocyte sizes in PGW from control or $Cdkn2a^{Ucp1}$ KO male mice (n = 3).

k, Representative H&E staining images of liver from control or $Cdkn2a^{Ucp1}$ KO male mice. Scale bar, 100 μM.

l, Weight of adipose tissues and other tissues from control or $Cdkn2a^{Ucp1}$ KO male mice (n = 7).

P values were determined by two-tailed Student's t test. n = 6-8 mice per group. Data are expressed as means ± SEM.

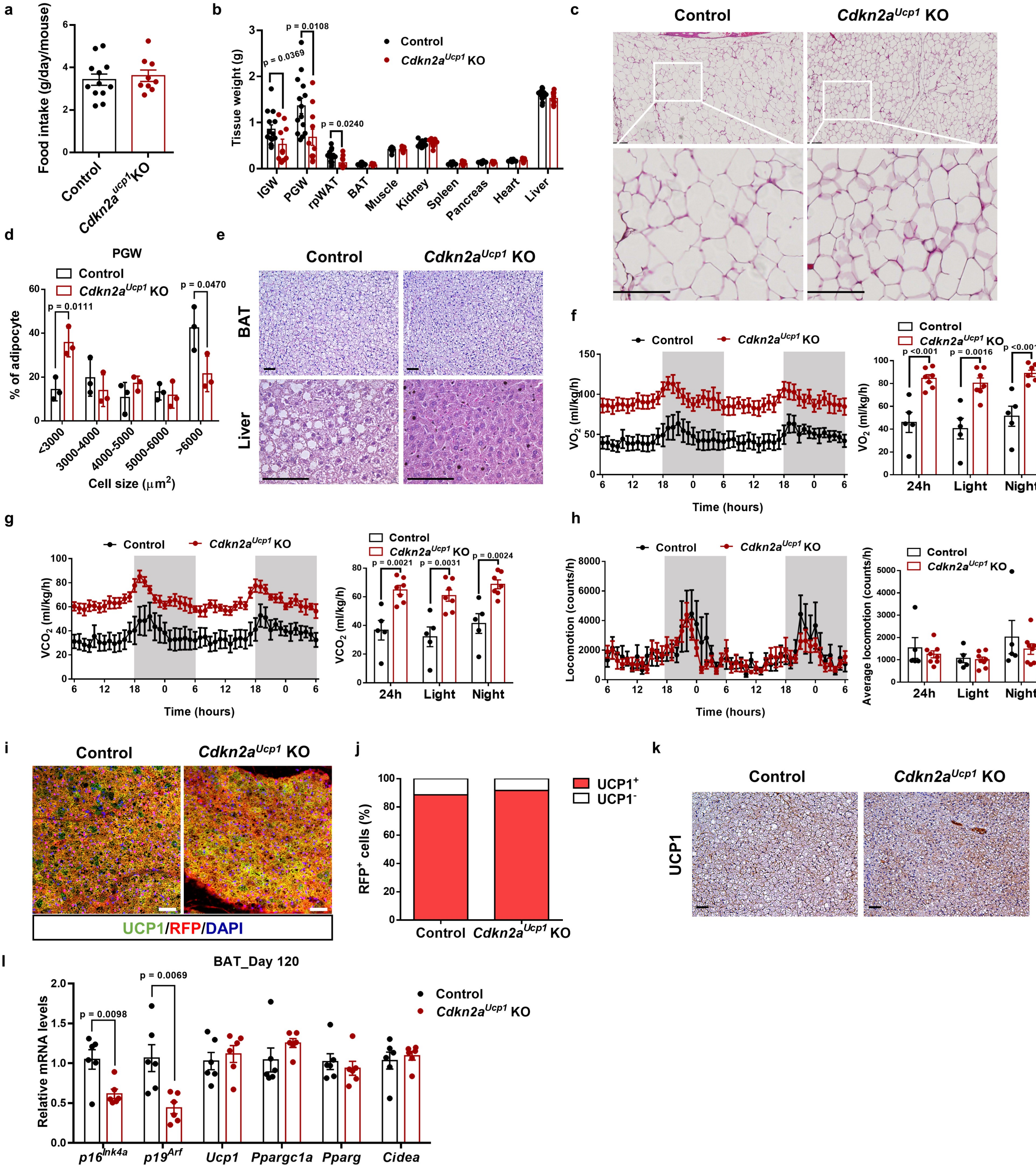

a
Food intake (g/day/mouse)
Control
Cdkn2a Ucp1 KO
b
Tissue weight (g)
p = 0.0369
p = 0.0108
p = 0.0240
Control
Cdkn2a Ucp1 KO
IGW
PGW
rpWAT
BAT
Muscle
Kidney
Spleen
Pancreas
Heart
Liver
c
Control
Cdkn2a Ucp1 KO
d
PGW
Control
Cdkn2a Ucp1 KO
p = 0.0111
p = 0.0470
% of adipocyte
<3000
3000-4000
4000-5000
5000-6000
>6000
Cell size (μm²)
e
Control
Cdkn2a Ucp1 KO
BAT
Liver
f
Control
Cdkn2a Ucp1 KO
VO2 (ml/kg/h)
Time (hours)
p <0.001
p = 0.0016
p <0.001
24h
Light
Night
g
Control
Cdkn2a Ucp1 KO
VCO2 (ml/kg/h)
Time (hours)
p = 0.0021
p = 0.0031
p = 0.0024
24h
Light
Night
h
Control
Cdkn2a Ucp1 KO
Locomotion (counts/h)
Time (hours)
Average locomotion (counts/h)
24h
Light
Night
i
Control
Cdkn2a Ucp1 KO
UCP1/RFP/DAPI
j
RFP+ cells (%)
UCP1+
UCP1-
Control
Cdkn2a Ucp1 KO
k
Control
Cdkn2a Ucp1 KO
UCP1
l
BAT_Day 120
Relative mRNA levels
p = 0.0098
p = 0.0069
Control
Cdkn2a Ucp1 KO
p16 Ink4a
p19 Arf
Ucp1
Ppargc1a
Pparg
Cidea

Supplementary Fig. 2: *Cdkn2a* deficiency has no effect on BAT thermogenesis in mice under HFHS.

a, Food intake of control (n = 12) or *Cdkn2a*^{Ucp1} KO (n = 9) male mice under HFHS.

b, Weight of adipose tissues and other tissues from control (n = 12-14) or *Cdkn2a*^{Ucp1} KO (n = 10-11) male mice under HFHS.

c, Tile scan of PGW from control or *Cdkn2a*^{Ucp1} KO male mice under HFHS. Scale bar, 100 μM.

d, Quantification of adipocyte sizes in PGW from control or *Cdkn2a*^{Ucp1} KO male mice (n = 3).

e, Representative H&E staining images of BAT and liver from control or *Cdkn2a*^{Ucp1} KO male mice under HFHS. Scale bar, 100 μM.

f, O_2 consumption of control (n = 5) or *Cdkn2a*^{Ucp1} KO (n = 7) male mice fed a HFHS. White and gray areas in the graphs indicate light and night, respectively.

g, CO_2 generation of control (n = 5) or *Cdkn2a*^{Ucp1} KO (n = 7) male mice fed a HFHS.

h, Locomotor activity of control (n = 5) or *Cdkn2a*^{Ucp1} KO (n = 7) male mice fed a HFHS.

i, Immunofluorescence staining of UCP1 and RFP in BAT from control or *Cdkn2a*^{Ucp1} KO male mice under HFHS. Scale bar, 50 μM.

j, Quantification of the percentage of RFP^{+} cells that express endogenous UCP1.

k, Immunohistochemical detection of UCP1 in IGW from control or *Cdkn2a*^{Ucp1} KO male mice under HFHS. Scale bar, 100 μM.

l, qPCR analysis of mRNA expression of thermogenic genes in BAT from control or *Cdkn2a*^{Ucp1} KO male mice under HFHS at day 120 post withdrawal cold stimulus (n = 6).

P values were determined by two-tailed Student's t test. n = 6-8 mice per group. Data are expressed as means ± SEM.

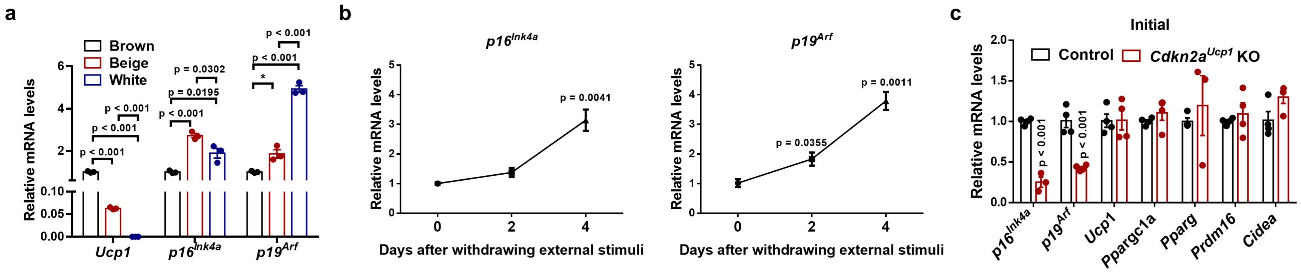

a
Brown
Beige
White
Relative mRNA levels
Ucp1
p16Ink4a
p19Arf
p < 0.001
p = 0.0302
p = 0.0195
*
b
p16Ink4a
p19Arf
Relative mRNA levels
p = 0.0041
p = 0.0355
p = 0.0011
Days after withdrawing external stimuli
c
Initial
Control
Cdkn2aUcp1 KO
Relative mRNA levels
p < 0.001
p16Ink4a
p19Arf
Ucp1
Ppargc1a
Pparg
Prdm16
Cidea

Supplementary Fig. 3: *Cdkn2a* is associated with beige adipocyte maintenance.

a, qPCR analysis of the mRNA levels of $p16^{Ink4a}$ and $p19^{Arf}$ in brown, beige and white adipocytes.

b, Expression profile of $p16^{Ink4a}$ and $p19^{Arf}$ during beige adipocyte maintenance.

c, qPCR analysis of mRNA expression of $p16^{Ink4a}$, $p19^{Arf}$ and thermogenic genes in control and $Cdkn2a^{Ucp1}$ KO beige adipocytes before withdrawing external stimuli (n = 3-4).

P values were determined by two-tailed Student's t test. Data are expressed as means ± SEM of triplicate tests.

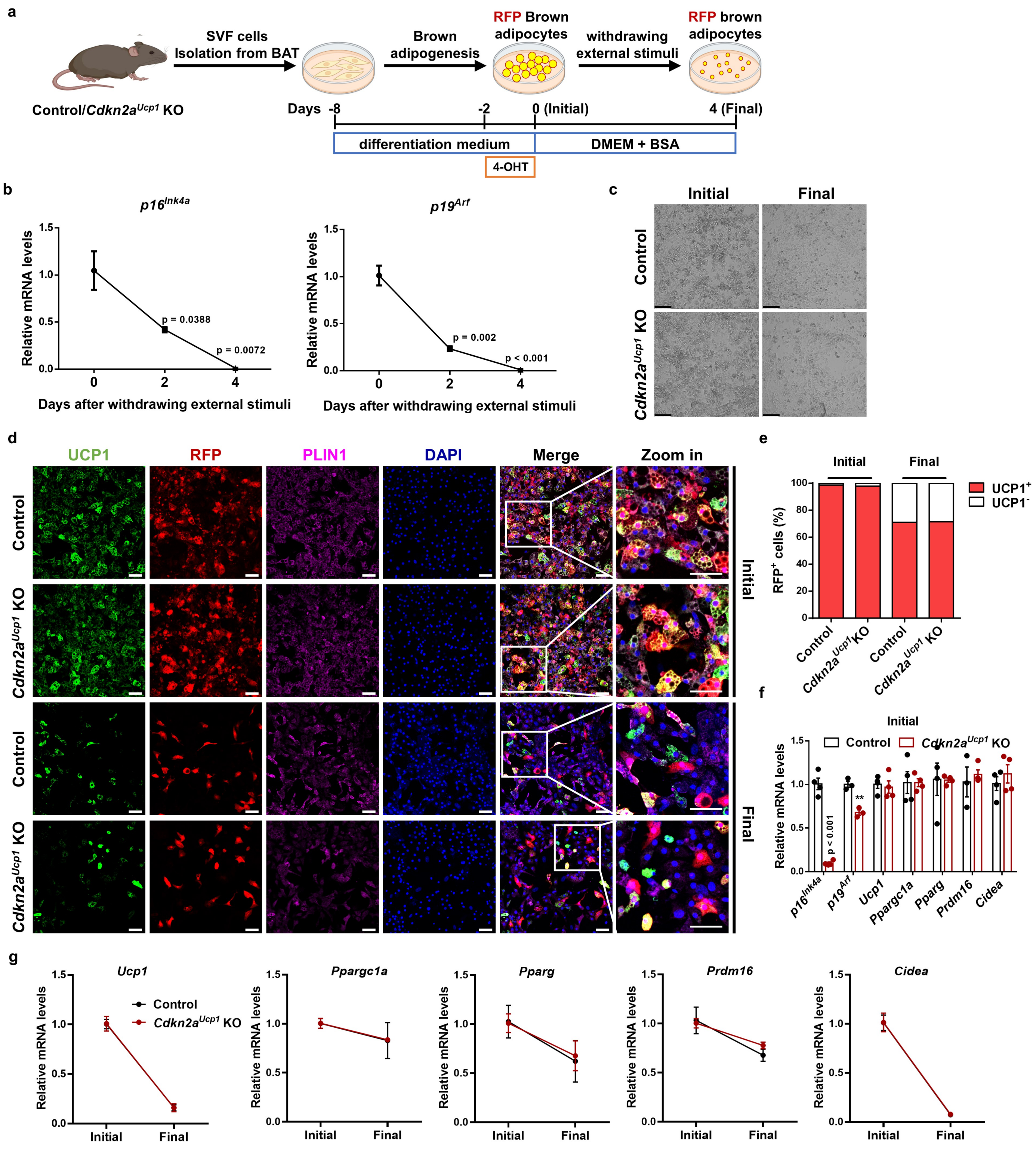

a
SVF cells
Isolation from BAT
Brown
adipogenesis
RFP Brown
adipocytes
withdrawing
external stimuli
RFP brown
adipocytes
Control/Cdkn2aUcp1 KO
Days -8
-2
0 (Initial)
4 (Final)
differentiation medium
DMEM + BSA
4-OHT
b
p16Ink4a
p19Arf
Relative mRNA levels
p = 0.0388
p = 0.0072
p = 0.002
p < 0.001
Days after withdrawing external stimuli
c
Initial
Final
Control
Cdkn2aUcp1 KO
d
UCP1
RFP
PLIN1
DAPI
Merge
Zoom in
Initial
Final
e
UCP1+
UCP1-
RFP+ cells (%)
f
Initial
Control
Cdkn2aUcp1 KO
p < 0.001
**
p16Ink4a
p19Arf
Ucp1
Ppargc1a
Pparg
Prdm16
Cidea
g
Ucp1
Ppargc1a
Pparg
Prdm16
Cidea
Control
Cdkn2aUcp1 KO
Initial
Final

Supplementary Fig. 4: *Cdkn2a* knockout does not affect brown fat maintenance *in vitro.*

a, Schematic illustration of the cellular system of brown adipocyte maintenance. SVF cells isolated from BAT of control or *Cdkn2a*^{Ucp1} KO male mice were differentiated into brown adipocytes and treated with 4-OHT to induce gene deletion and RFP labeling. Then, external stimuli were withdrawn by changing the differentiation medium to DMEM containing 5% BSA for 4 days.

b, Expression profile of *p16*^{Ink4a} and *p19*^{Arf} during brown adipocyte maintenance.

c, Bright field images of control and *Cdkn2a*^{Ucp1} KO brown adipocytes during brown adipocyte maintenance. Scale bar, 100 μM.

d, Immunofluorescence staining of UCP1 and RFP in control and *Cdkn2a*^{Ucp1} KO brown adipocytes during brown adipocyte maintenance. Scale bar, 50 μM.

e, Quantification of the percentage of RFP+ cells that express endogenous UCP1.

f, qPCR analysis of the mRNA expression of *p16*^{Ink4a}, *p19*^{Arf}, and thermogenic genes in control and *Cdkn2a*^{Ucp1} KO brown adipocytes before withdrawing external stimuli (n = 3-4).

g, qPCR analysis of the mRNA expression of thermogenic genes in control and *Cdkn2a*^{Ucp1} KO brown adipocytes during brown adipocyte maintenance.

P values were determined by two-tailed Student’s t test. Data are expressed as means ± SEM of triplicate tests.

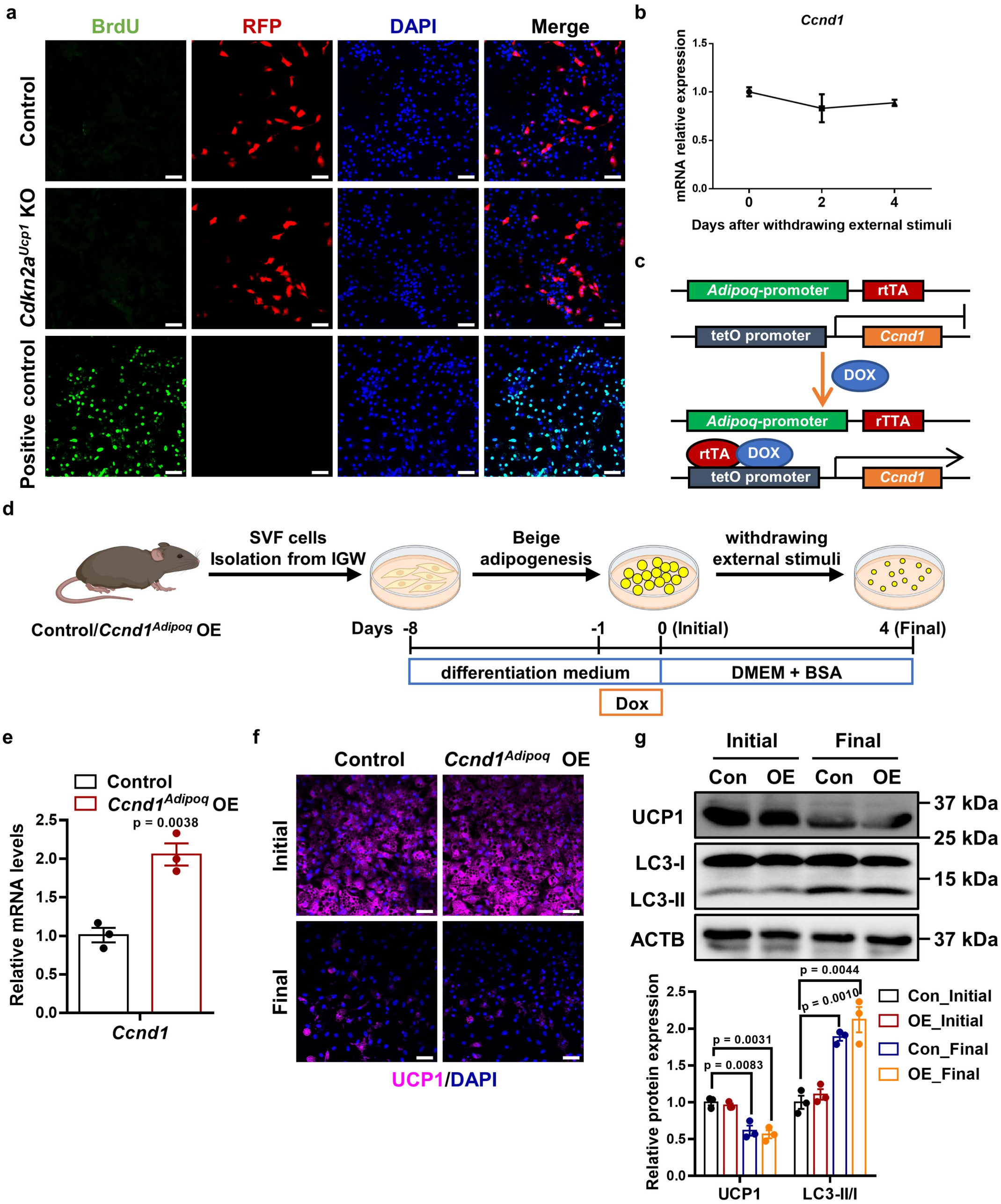

a
BrdU
RFP
DAPI
Merge
Control
Cdkn2a Ucp1 KO
Positive control
b
Ccnd1
mRNA relative expression
Days after withdrawing external stimuli
c
Adipoq-promoter
rtTA
tetO promoter
Ccnd1
DOX
d
SVF cells
Isolation from IGW
Beige
adipogenesis
withdrawing
external stimuli
Control/Ccnd1 Adipoq OE
Days -8
-1
0 (Initial)
4 (Final)
differentiation medium
DMEM + BSA
Dox
e
Control
Ccnd1 Adipoq OE
p = 0.0038
Relative mRNA levels
Ccnd1
f
Control
Ccnd1 Adipoq OE
Initial
Final
UCP1/DAPI
g
Initial
Final
Con
OE
UCP1
LC3-I
LC3-II
ACTB
37 kDa
25 kDa
15 kDa
p = 0.0044
p = 0.0010
p = 0.0031
p = 0.0083
Relative protein expression
UCP1
LC3-II/I
Con_Initial
OE_Initial
Con_Final
OE_Final

Supplementary Fig. 5: CCND1 does not regulate beige adipocyte maintenance.

a, Immunofluorescence staining of BrdU and RFP in control and *Cdkn2a*^{Ucp1} KO beige adipocytes after withdrawing external stimuli. Scale bar, 100 μM.

b, Expression profile of *Ccnd1* during beige adipocyte maintenance.

c, Scheme of *Adipoq* promoter-driven Dox-inducible *Ccnd1* transgenic mice.

d, Schematic illustration of the cellular system. SVF cells isolated from IGW of control or *Ccnd1*^{Adipoq} OE male mice were differentiated into beige adipocytes and treated with doxycycline (Dox) to induce gene overexpression. Then, external stimuli were withdrawn by changing the differentiation medium to DMEM containing 5% BSA for 4 days.

e, qPCR analysis of mRNA levels of *Ccnd1* in control and *Ccnd1*^{Adipoq} OE beige adipocytes.

f, Immunofluorescence staining of UCP1 in control and *Ccnd1*^{Adipoq} OE beige adipocytes during beige adipocyte maintenance. Scale bar, 50 μM.

g, Western blot analysis of UCP1 and LC3 in control and *Ccnd1*^{Adipoq} OE beige adipocytes during beige adipocyte maintenance.

P values were determined by two-tailed Student's t test. Data are expressed as means ± SEM of triplicate tests.

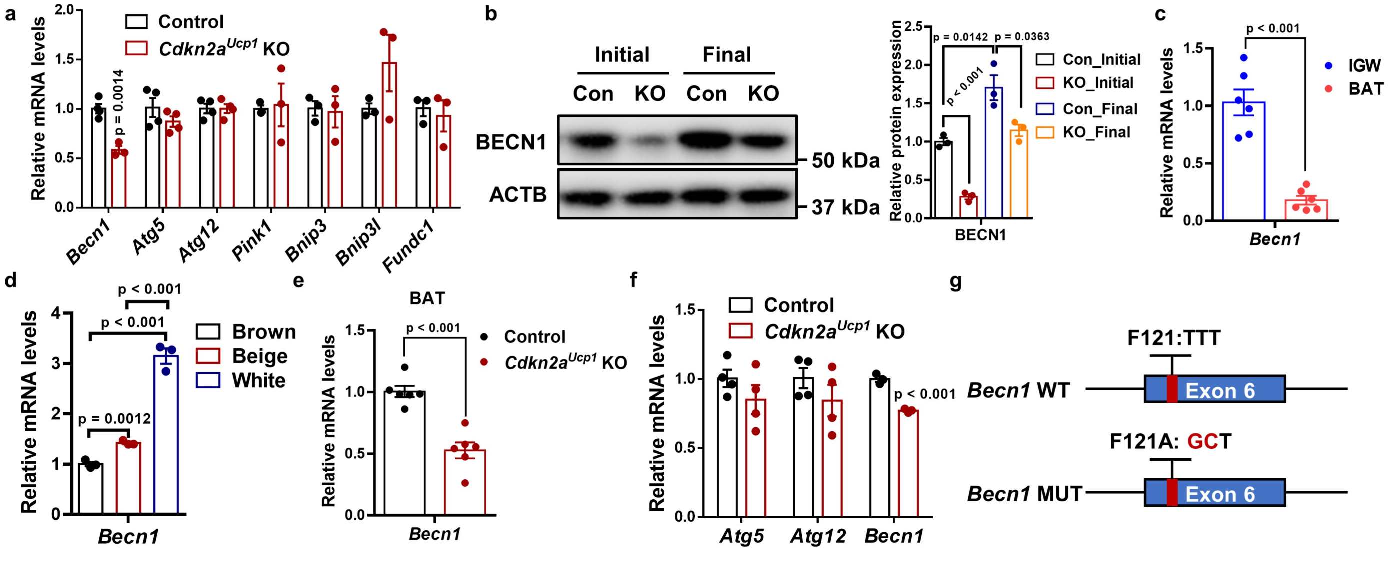

a
Control
Cdkn2a^Ucp1 KO
Relative mRNA levels
p = 0.0014
Becn1
Atg5
Atg12
Pink1
Bnip3
Bnip3l
Fundc1
b
Initial
Final
Con
KO
Con
KO
BECN1
ACTB
50 kDa
37 kDa
Relative protein expression
p = 0.0142
p = 0.0363
p < 0.001
Con_Initial
KO_Initial
Con_Final
KO_Final
BECN1
c
p < 0.001
Relative mRNA levels
IGW
BAT
Becn1
d
Relative mRNA levels
p < 0.001
p < 0.001
p = 0.0012
Brown
Beige
White
Becn1
e
BAT
p < 0.001
Relative mRNA levels
Control
Cdkn2a^Ucp1 KO
Becn1
f
Control
Cdkn2a^Ucp1 KO
Relative mRNA levels
p < 0.001
Atg5
Atg12
Becn1
g
F121:TTT
Becn1 WT
Exon 6
F121A: GCT
Becn1 MUT
Exon 6

Supplementary Fig. 6: Becn1 is a potential regulator of beige fat maintenance.

a, qPCR analysis of the mRNA expression of autophagy-related genes in control and *Cdkn2a*^{Ucp1} KO beige adipocytes after withdrawing external stimuli.

b, Western blot analysis of BECN1 in control and *Cdkn2a*^{Ucp1} KO beige adipocytes after withdrawing external stimuli.

c, qPCR analysis of the mRNA levels of *Becn1* in IGW or BAT from wild type male mice (n = 6).

d, qPCR analysis of the mRNA levels of *Becn1* in brown, beige, and white adipocytes.

e, qPCR analysis of the mRNA expression of *Becn1* in BAT from control or *Cdkn2a*^{Ucp1} KO male mice (n = 6).

f, qPCR analysis of *Atg5*, *Atg7* and *Becn1* mRNA expression in control and *Cdkn2a*^{Ucp1} KO brown adipocytes after withdrawal of external stimuli (n = 3-4).

g, Scheme of the autophagy-hyperactive *Becn1*^{F121A} knock-in mouse model.

P values were determined by two-tailed Student's t test. Data are expressed as means ± SEM of triplicate tests.

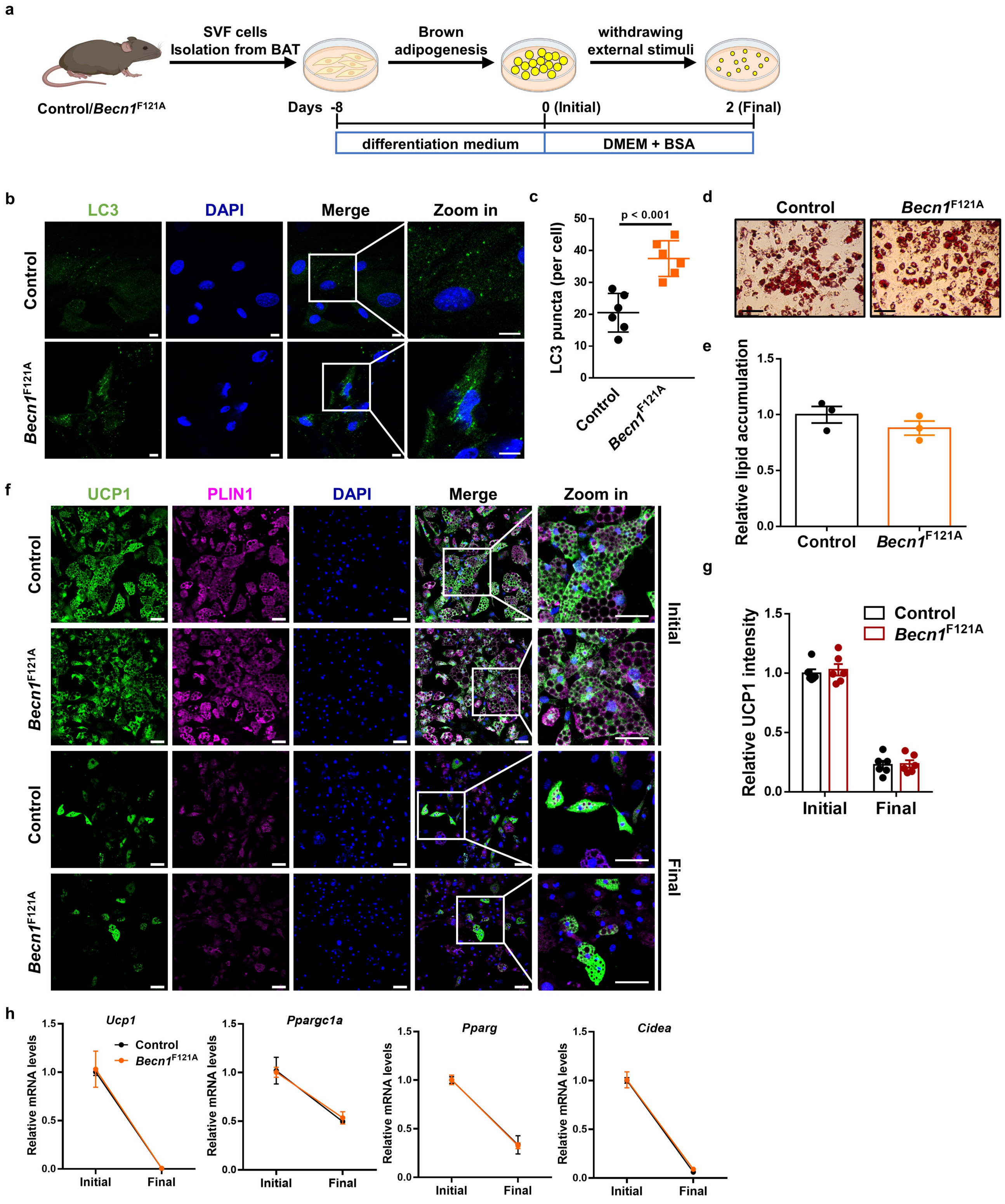
a
SVF cells
Isolation from BAT
Brown
adipogenesis
withdrawing
external stimuli
Control/Becn1F121A
Days -8
0 (Initial)
2 (Final)
differentiation medium
DMEM + BSA
b
LC3
DAPI
Merge
Zoom in
Control
Becn1F121A
c
p < 0.001
LC3 puncta (per cell)
Control
Becn1F121A
d
Control
Becn1F121A
e
Relative lipid accumulation
Control
Becn1F121A
f
UCP1
PLIN1
DAPI
Merge
Zoom in
Control
Becn1F121A
Initial
Final
g
Relative UCP1 intensity
Control
Becn1F121A
Initial
Final
h
Ucp1
Ppargc1a
Pparg
Cidea
Relative mRNA levels
Control
Becn1F121A
Initial
Final

Supplementary Fig. 7: Hyperactive *Becn1*^{F121A} does not influence brown adipocyte differentiation and maintenance.

a, Schematic illustration of the cellular system of brown adipocyte maintenance. SVF cells isolated from BAT of control or *Becn1*^{F121A} male mice were differentiated into brown adipocytes. Then, external stimuli were withdrawn by changing the differentiation medium to DMEM containing 5% BSA for 2 days.

b, Immunofluorescence staining of LC3 in control and *Becn1*^{F121A} brown adipocytes. Scale bar, 10 μM.

c, Quantification of the number of LC3 puncta per cell (n = 6).

d, Oil red O staining of control and *Becn1*^{F121A} brown adipocytes before withdrawing external stimuli. Scale bar, 100 μM.

e, Relative lipid accumulation was quantified with a microplate spectrophotometer.

f, Immunofluorescence staining of UCP1 and PLIN1 in control and *Becn1*^{F121A} brown adipocytes during brown adipocyte maintenance. Scale bar, 50 μM.

g, Quantification of the percentage of UCP1-expressing cells (n = 6).

h, qPCR analysis of mRNA expression of thermogenic genes in control and *Becn1*^{F121A} brown adipocytes after withdrawal of external stimuli.

P values were determined by two-tailed Student's t test. Data are expressed as means ± SEM of triplicate tests.

Supplementary Tables

Supplementary Table 1. The information of antibodies used in this study.

Antibodies	Source	Identifier	Dilutions
Rabbit anti-UCP1	Thermo Fisher Scientific	Cat# PA1-24894	1:1000 for WB; 1:200 for IF
Mouse anti-RFP	Takara	Cat# 632392	1:300 for IF
Rabbit anti-ACTB	Cell Signaling Technology	Cat# 4970	1:1000 for WB
Rabbit anti-TH	Abcam	Cat# ab75875	1:1000 for WB
Rabbit anti-VDAC	Thermo Fisher Scientific	Cat# PA1-954A	1:1000 for WB
Rabbit anti-TOMM20	Thermo Fisher Scientific	Cat# PA5-52843	1:1000 for WB
Rabbit anti-BCL2L1	Cell Signaling Technology	Cat# 2764	1:1000 for WB; 1:100 for IP
Rabbit anti-BECN1	Thermo Fisher Scientific	Cat# PA1-16857	1:1000 for WB
Rabbit anti-LC3A/B	Cell Signaling Technology	Cat# 12741	1:1000 for WB; 1:100 for IF
Rabbit anti-p14ARF	Novus Biologicals	Cat# NB200-111	1:200 for IF; 1:500 for IP
Mouse anti-p16INK4a	Santa Cruz	Cat# sc-1661	1:200 for IF; 1:100 for IP
Rabbit anti-Gamma Globulin	Thermo Fisher Scientific	Cat# 31887	1:5000 for IP
Rabbit anti-SQSTM1/P62	Thermo Fisher Scientific	Cat# PA5-20839	1:1000 for WB
Goat anti-Perilipin	Abcam	Cat#: ab61682	1:250 for IF
Mouse anti-BrdU	DSHB	Cat#: G3G4	1:200 for IF
HRP goat anti-rabbit	Cell Signaling Technology	Cat#: 96714S	1:3000 for WB
HRP goat anti-mouse	Cell Signaling Technology	Cat#: 7074S	1:3000 for WB
cy3 donkey anti-mouse	Jackson ImmunoResearch	Cat#: 715-165-150	1:400 for IF
cy3 goat anti-rabbit	Jackson ImmunoResearch	Cat#: 111-165-003	1:400 for IF
AF488 donkey anti-rabbit	Jackson ImmunoResearch	Cat#: 711-545-152	1:400 for IF
AF488 donkey anti-mouse	Jackson ImmunoResearch	Cat#: 715-545-150	1:400 for IF
cy5 donkey anti-rat	Jackson ImmunoResearch	Cat#: 712-175-153	1:400 for IF
cy5 donkey anti-goat	Jackson ImmunoResearch	Cat#: 705-175-147	1:400 for IF

Supplementary Tables

Supplementary Table 2. Sequences of primers.

**Gene**	**Species**	**Forward primer (5'-3')**	**Reverse primer (5'-3')**
Adipoq	Mouse	GCAGGCATCCCAGGACATC	GCGATACATATAAGCGGCTTCT
Atg5	Mouse	ATGCGGTTGAGGCTCACTTTA	GGTTGATGGCCCAAAACTGG
Atg12	Mouse	TGTGAATCAGTCCTTTGCCCC	TGCAGGACCAGTTTACCATCAC
Becn1	Mouse	ATGCAGGTGAGCTTCGTGTG	AATGGCTCCTGTGAGTTCCTG
Bnip3	Mouse	TCCTGGGTAGAACTGCACTTC	GCTGGGCATCCAACAGTATTT
Bnip3l	Mouse	TGTCTCACTTAGTCGAGCCGC	TGGGTAGCTCCACCCAGGAA
Cdkn2a (p16^{Ink4a})	Mouse	CGAACTCTTTCGGTCGTAC	ATCATCATCACCTGAATCGGGGT
Cdkn2a (p19^{Arf})	Mouse	GCAGGTTCTTGGTCACTGT	TCGCACGAACTTCACC
Cidea	Mouse	TGACATTCATGGGATTGCAGAC	GGCCAGTTGTGATGACTAAGAC
Cox2	Mouse	AGTTGATAACCGAGTCGTTCTG	CTGTTGCTTGATTTAGTCGGC
Fabp4	Mouse	CTGGGCGTGGAATTCGAT	GCTCTTCACCTTCCTGTCGTCT
Fundc1	Mouse	ACCATAGACTTCCAACTCGGC	TTCCGGGATGCCATGATACC
Hbb	Mouse	GAAGCGATTCTAGGGAGCAG	GGAGCAGCGATTCTGAGTAGA
Leptin	Mouse	AAGACCATTGTCACCAGGATCAA	GGATACCGACTGCGTGTGTG

Pink1	Mouse	CCACCTTTCCCTTTGCCATC	ACTGCTCCATACTCTCCAGC
Prdm16	Mouse	ACACGCCAGTTCTCCAACCTGT	TGCTTGTTGAGGGAGGAGGTA
Pparg	Mouse	GCATGGTGCCTTCGCTGA	TGGCATCTCTGTGTCAACCATG
Ppargc1a	Mouse	CCGATCACCATATTCCAGGT	GTGTGCGGTGTCTGTAGTGG
Rrlp0	Mouse	TCCAGGCTTTGGGCATCA	CTTTATCAGCTGCACATCACTCAGA
Ucp1	Mouse	CACCTTCCCGCTGGACACT	CCCTAGGACACCTTTATACCTAATGG
ACTB	Human	GAGCTACGAGCTGCCTGACG	GTAGTTTCGTGGATGCCACAG
BECN1	Human	TGCCGTTATACTGTTCTGGGG	CGTGTCTCGCCTTTCTCAAC
CIDEA	Human	TTATGGGATCACAGACTAAGCGA	TGCTCCTGTCATGGTTGGAGA
CDKN2A ($p16^{Ink4a}$)	Human	GGGGTCGGGTAGAGGAGG	TCATCATGACCTGGATCGGC
CDKN2A ($p14^{Arf}$)	Human	GGTTCTTGGTGACCCTCCG	TCAGTAGCATCAGCACGAGG
PPARGC1A	Human	TCTGAGTCTGTATGGAGTGACAT	CCAAGTCGTTCACATCTAGTTCA
PRDM16	Human	CTTCGGATGGGAGCAAATACTG	TCCACGCAGAACTTCTCACTG
UCP1	Human	AGAAGGGCGGATGAAACTCT	ATCCTGGACCGTGTCGTAG

Supplementary Tables

Supplementary Table 3. The details of population characteristics.

**Group name**	**Sex, female/male (n)**	**Age**	**Body weight (Kg)**	**BMI**	**Fat mass (Kg)**	**Fasting plasma glucose (mg/dl)**
Non-obese	3/2	37.40±2.857	61.79±5.817	22.26±1.240	18.56±1.293	85.60±3.816
Obese	9/3	36.08±2.773	142.9±8.424	51.00±2.119	69.61±5.565	113.2±8.302

Data indicate means ± SEM
