# Supplementary material for: Genetically prolonged beige fat in male mice confers long-lasting metabolic health
Source: Nat Commun. 2023 May 12;14:2731. doi: 10.1038/s41467-023-38471-z (PMC10175245; doi:10.1038/s41467-023-38471-z)
Supplement: Supplementary file 3 — Reporting Summary [file 41467_2023_38471_MOESM3_ESM.pdf]

nature portfolio

Corresponding author(s): Yuwei Jiang
Last updated by author(s): Apr 18, 2023

Reporting Summary

Nature Portfolio wishes to improve the reproducibility of the work that we publish. This form provides structure for consistency and transparency in reporting. For further information on Nature Portfolio policies, see our Editorial Policies and the Editorial Policy Checklist.

Statistics

For all statistical analyses, confirm that the following items are present in the figure legend, table legend, main text, or Methods section.

n/a	Confirmed	
☐	☒	The exact sample size (*n*) for each experimental group/condition, given as a discrete number and unit of measurement
☐	☒	A statement on whether measurements were taken from distinct samples or whether the same sample was measured repeatedly
☐	☒	The statistical test(s) used AND whether they are one- or two-sided<br>*Only common tests should be described solely by name; describe more complex techniques in the Methods section.*
☐	☒	A description of all covariates tested
☒	☐	A description of any assumptions or corrections, such as tests of normality and adjustment for multiple comparisons
☐	☒	A full description of the statistical parameters including central tendency (e.g. means) or other basic estimates (e.g. regression coefficient) AND variation (e.g. standard deviation) or associated estimates of uncertainty (e.g. confidence intervals)
☐	☒	For null hypothesis testing, the test statistic (e.g. *F*, *t*, *r*) with confidence intervals, effect sizes, degrees of freedom and *P* value noted<br>*Give P values as exact values whenever suitable.*
☒	☐	For Bayesian analysis, information on the choice of priors and Markov chain Monte Carlo settings
☒	☐	For hierarchical and complex designs, identification of the appropriate level for tests and full reporting of outcomes
☐	☒	Estimates of effect sizes (e.g. Cohen's *d*, Pearson's *r*), indicating how they were calculated

Our web collection on statistics for biologists contains articles on many of the points above.

Software and code

Policy information about availability of computer code

Data collection	Bruker Minispec 10 whole composition analyzer was used to collect body composition in mice.<br>Via7 System was used to collect real time PCR data.<br>Images of both H&E and Immunostaining were collected by Leica DMi8 microscope and confocal laser microscope.<br>Metabolic cage data were collected by Promethion System.<br>Oxygen consumption rate of stromal vascular fraction cells were collected by Seahorse XFe96 Extracellular Flux Analyzer.<br>Glucose levels were collected by Contour Next glucose meter.
Data analysis	All data were statistically analyzed in GradhPad Prism 9. All images were analyzed by NIH Image J software 1.51. All data were coordinated in Excel of Office 365

For manuscripts utilizing custom algorithms or software that are central to the research but not yet described in published literature, software must be made available to editors and reviewers. We strongly encourage code deposition in a community repository (e.g. GitHub). See the Nature Portfolio guidelines for submitting code & software for further information.

Data

Policy information about availability of data

All manuscripts must include a data availability statement. This statement should provide the following information, where applicable:

- Accession codes, unique identifiers, or web links for publicly available datasets
- A description of any restrictions on data availability
- For clinical datasets or third party data, please ensure that the statement adheres to our policy

The data that support the findings of this study are available in the methods and supplementary material of this article. Source data are provided with this paper. Additional data are available from the corresponding author on reasonable request.

Human research participants

Policy information about studies involving human research participants and Sex and Gender in Research.

Reporting on sex and gender	We enrolled twelve obese subjects (8 females and 4 males) who underwent laparoscopic gastric sleeve bariatric surgery and five lean healthy controls (3 females and 2 males) who underwent elective surgeries such as hernia repair and abdominal wall reconstruction.The age of the participants ranged from 21 to 47 years old.
Population characteristics	Full details of population characteristics are listed in Table 3.
Recruitment	Study participants were recruited from the Bariatric and General Surgery Clinics at the University of Illinois Hospital. Exclusion criteria included chronic inflammatory diseases, chronic organ failure, autoimmune diseases, cancer of any type, current smoking, or current pregnancy. Subjects who were deemed eligible were informed about the study details, risks, and precautions taken to reduce this risk. All the study participants provided informed written consent.<br><br>To reduce the risk of selection bias, subjects are recruited from the Greater Chicago area using both active and passive recruitment strategies. For passive recruitment, flyers are distributed throughout the communities surrounding UIC, UI Health clinical areas, and via electronic announcements. For active recruitment, the eligibility criteria for selecting patients who are scheduled for bariatric or elective surgery are communicated to healthcare providers. Bariatric surgery patients are also recruited at the Nutrition and Wellness Center and the Bariatric Surgery Clinic, where they are seen prior to and after surgery. The PI of the study systematically evaluates patients for eligibility, and those who agree to participate and meet all criteria provide written consent.
Ethics oversight	All protocols and procedures of the study followed the standards set by the latest version of the Declaration of Helsinki and were approved by the Institutional Review Board of The University of Illinois at Chicago (protocol code 2017-1125).

Note that full information on the approval of the study protocol must also be provided in the manuscript.

Field-specific reporting

Please select the one below that is the best fit for your research. If you are not sure, read the appropriate sections before making your selection.

☒ Life sciences ☐ Behavioural & social sciences ☐ Ecological, evolutionary & environmental sciences

For a reference copy of the document with all sections, see nature.com/documents/nr-reporting-summary-flat.pdf

Life sciences study design

All studies must disclose on these points even when the disclosure is negative.

Sample size	For all experiments, we chose a minimum sample size of 3, and each experiment was conducted twice. Sample size determination is based on the previous experience to obtain significance and reproducibility (Jiang Y, et al, Nature Communications, 2017, PMID: 28649987; Park J, et al, Developmental Cell 2021, PMID: 33711247), as well as minimizing the number of animals used as required by the animal ethics committee. The sample size following common standards employing three or more biological replicates. All sample sizes are listed in each figure legend.
Data exclusions	All data collected for this study were included and analyzed, and no data were excluded.
Replication	Three biological replicates were used for verifying the reproducibility of the experimental findings in both mouse experiments and cell culture experiments. All the attempts at replication were successful. We have added the statement in the Figure legends and the source data in the source data file.
Randomization	In order to minimize the influence of individual differences in mice, we use littermates for experiments. Littermates were randomly allocated

	into experimental groups.
Blinding	Although we were unable to blind the experimental design and execution of both in vivo and in vitro experiments due to a lack of trained personnel in the lab, we ensured that all data analysis and image quantification were conducted in a blinded manner.

Reporting for specific materials, systems and methods

We require information from authors about some types of materials, experimental systems and methods used in many studies. Here, indicate whether each material, system or method listed is relevant to your study. If you are not sure if a list item applies to your research, read the appropriate section before selecting a response.

Materials & experimental systems

n/a	Involved in the study	
☐	☒	Antibodies
☒	☐	Eukaryotic cell lines
☒	☐	Palaeontology and archaeology
☐	☒	Animals and other organisms
☒	☐	Clinical data
☒	☐	Dual use research of concern

Methods

n/a	Involved in the study	
☒	☐	ChIP-seq
☒	☐	Flow cytometry
☒	☐	MRI-based neuroimaging

Antibodies

Antibodies used

antibodies used in this study as follows:
Rabbit anti-UCP1 Thermo Fisher Scientific Cat# PA1-24894
Mouse anti-RFP Takara Cat# 632392
Rabbit anti-ACTB Cell Signaling Technology Cat# 4970
Rabbit anti-BCL2L1 Cell Signaling Technology Cat# 2764
Rabbit anti-BECN1 Thermo Fisher Scientific Cat# PA1-16857
Rabbit anti-LC3A/B Cell Signaling Technology Cat# 12741
Rabbit anti-p14ARF Novus Biologicals Cat# NB200-111
Mouse anti-p16INK4a Santa Cruz Cat# sc-1661
Rabbit anti-Gamma Globulin Thermo Fisher Scientific Cat# 31887
Rabbit anti-SQSTM1/P62 Thermo Fisher Scientific Cat# PA5-20839
Rabbit anti-TH Abcam Cat#: ab75875
Rabbit anti-VDAC Thermo Fisher Scientific Cat# PA1-954A
Rabbit anti-TOMM20 Thermo Fisher Scientific Cat# PA5-52843
Goat anti-Perilipin Abcam Cat#: ab61682
Mouse anti-BrdU DSHB Cat#: G3G4
HRP goat anti-rabbit Cell Signaling Technology Cat#: 96714S
HRP goat anti-mouse Cell Signaling Technology Cat#: 7074S
cy3 donkey anti-mouse Jackson ImmunoResearch Cat#: 715-165-150
cy3 goat anti-rabbit Jackson ImmunoResearch Cat#: 111-165-003
AF488 donkey anti-rabbit Jackson ImmunoResearch Cat#: 711-545-152
AF488 donkey anti-mouse Jackson ImmunoResearch Cat#: 715-545-150
cy5 donkey anti-rat Jackson ImmunoResearch Cat#: 712-175-153
cy5 donkey anti-goat Jackson ImmunoResearch Cat#: 705-175-147

Validation	For this study, we used antibodies that were validated based on the provided data sheets and references for the specific technique used (western blot, immunostaining). All dilutions used in the experiments can be found within the methods section of the manuscript.<br>Clone name / Published Species / Applications<br>Rabbit-anti-UCP1 / Human, Mouse, Rat / WB, IHC, IP, IF<br>Mouse-anti-RFP /Human, Mouse, Rat / WB, IP, IF<br>Rabbit-anti-ACTB / Human, Mouse, Rat, Pig, Monkey / WB, IHC, IF<br>Rabbit-anti-BCL2L1 / Human, Mouse, Rat, Monkey / WB, IP, IHC, IF<br>Rabbit-anti-BECN1 / Human, Mouse, Rat / WB, IHC, IP, IF, Flow<br>Rabbit-anti-LC3A/B / Human, Mouse, Rat / WB, IHC, IF<br>Rabbit-anti-p14ARF / Human, Mouse / WB, IHC, IP, IF, Flow, Elisa<br>Mouse-anti-p16Ink4a / Human, Mouse / WB, IHC, IP, IF, Elisa<br>Rabbit-anti-Gamma Globulin / Human, Mouse, Rat / Flow, IP, Chip<br>Rabbit-anti-SQSTM/P62 / Human, Mouse, Rat / WB, IHC, IF<br>Goat-anti-Perilipin / Human, Mouse / WB, IHC, IF<br>Mouse-anti-BrdU / All species labeled with BrdU / IHC, IF, Flow

Animals and other research organisms

Policy information about studies involving animals; ARRIVE guidelines recommended for reporting animal research, and Sex and Gender in Research

Laboratory animals	All animal experiments were performed according to procedures reviewed and approved by the Institutional Animal Care and Use Committee of the University of Illinois at Chicago (protocol number 21-112). Rosa26RRFP (Stock No. 007914) mice were obtained from the Jackson Laboratory. Becn1F121A mice were generously provided by Dr. Congcong He (Northwestern University). Ucp1-CreERT2 and Cdkn2afl/fl mice were generously provided by Dr. Eric N. Olson (University of Texas Southwestern Medical Center) and were intercrossed for six generations prior to experimentation and were maintained on mixed C57BL6/J-129SV background. To induce recombination, denoted mice were administered one dose of TMX (1.5 mg/Kg; Cayman Chemical: 13258) dissolved in sunflower seed oil (Sigma, item no: S5007) for two consecutive days via intraperitoneal (IP) injection. For cold exposure experiments, mice were placed in a 6˚C environmental chamber (Environmental & Temperature Solutions) for 7 days or mice were maintained at RT (~23-25ºC; ~35% humidity). Body temperature was measured using a rectal probe (Physitemp). All animal experiments were performed on 3 or more male mice per cohort and performed at least twice. All experiments were performed on male mice at 2-, 3-, 4-, and 6-months of age.
Wild animals	No wild animals were used in the study
Reporting on sex	Male mice were used in this study
Field-collected samples	No field collected samples were used in the study
Ethics oversight	University of Illinois at Chicago Institutional Animal Care and Use Committee under the auspices of protocol number 21-112)

Note that full information on the approval of the study protocol must also be provided in the manuscript.
